# Visualization-Aided Classification Ensembles Discriminate Lung Adenocarcinoma and Squamous Cell Carcinoma Samples Using Their Gene Expression Profiles

**DOI:** 10.1371/journal.pone.0110052

**Published:** 2014-10-15

**Authors:** Ao Zhang, Chi Wang, Shiji Wang, Liang Li, Zhongmin Liu, Suyan Tian

**Affiliations:** 1 Intensive Care Unit (ICU), First Hospital of Jilin University, Changchun, Jilin, China; 2 Division of Clinical Epidemiology, First Hospital of Jilin University, Changchun, Jilin, China; 3 Department of Biostatistics and Markey Cancer Center, University of Kentucky, Lexington, Kentucky, United States of America; 4 LLX Solutions LLC., Waltham, Massachusetts, United States of America; University of North Carolina School of Medicine, United States of America

## Abstract

**Introduction:**

The widespread application of microarray experiments to cancer research is astounding including lung cancer, one of the most common fatal human tumors. Among non-small cell lung carcinoma (NSCLC), there are two major histological types of NSCLC, adenocarcinoma (AC) and squamous cell carcinoma (SCC).

**Results:**

In this paper, we proposed to integrate a visualization method called Radial Coordinate Visualization (Radviz) with a suitable classifier, aiming at discriminating two NSCLC subtypes using patients' gene expression profiles. Our analyses on simulated data and a real microarray dataset show that combining with a classification method, Radviz may play a role in selecting relevant features and ameliorating parsimony, while the final model suffers no or least loss of accuracy. Most importantly, a graphic representation is more easily understandable and implementable for a clinician than statistical methods and/or mathematic equations.

**Conclusion:**

To conclude, using the NSCLC microarray data presented here as a benchmark, the comprehensive understanding of the underlying mechanism associated with NSCLC and of the mechanisms with its subtypes and respective stages will become reality in the near future.

## Introduction

Lung cancer is one of the most common fatal human tumors, accounting for 25% of cancer death in both men and women throughout the world [1]. About 85% of lung cancers are non-small cell lung carcinoma (NSCLC), and two major histological types of NSCLC, adenocarcinoma (AC) and squamous cell carcinoma (SCC), represents about 40% of NSCLC cases, respectively [2].

In clinical practice, homogeneous treatment strategies have been traditionally implemented for both subtypes, which may explain the poor treatment response achieved in NSCLC. The recent-developed molecular targeted therapies such as an anti-epidermal growth factor receptor (EGFR) antibody are effective in patients harboring mutations in corresponding genes, which are exclusively found in AC. This indicates fundamental differences in the underlying mechanisms of tumor development, growth and invasion between the two subtypes. The prognosis of NSCLC also depends on tumor stage. The low survival rate is mainly attributable to late diagnosis, when the tumor has become unresectable. On the contrary, early-stage NSCLC patients have a significantly better prognosis. Therefore, the successful classification of NSCLC patients into their corresponding subtypes and stages is of clinical significance.

The widespread application of microarray experiments to cancer research is astounding. The lung cancer research is no exception. Many researchers have employed microarray technology to disclose the molecular nature of etiological differences in between these two NSCLC subtypes and/or their stages [3], [4]. For instance the recent SBV IMPROVER Diagnostics Signature Challenge [5], [6], a crowd-sourcing competition organized by Philip Morris International and IBM, aims at assessing and verifying computational approaches to the classification of clinical samples based on microarray data. One of the tasks, the lung cancer sub-challenge, aimed at to classify AC and SCC, and early stages (I and II) of these two histology stages using high-throughput gene expression data. The results from this sub-challenge indicated only the subtype segregation had been successfully achieved [7].

In this subtask, Tian and Suarez-Farinas proposed a regularization method called hierarchical- Threshold Gradient Decent Regularization (TGDR) [8] that utilized biological hierarchy and combined such internal structure with TGDR [9], a feature selection algorithm, to do parameter estimation and class-membership prediction on new samples. The detailed descriptions on TGDR were presented by Ma and Huang [10]. Although TGDR is an outstanding regularization algorithm with many excellent features, it is criticized for having inferior parsimony [11]. As an extension to TGDR, hierarchical-TGDR inherits this disadvantage. Previously, Tian and Suarez-Farinas [12] tackled this problem usingbagging procedure [13], which can reduce the number of false negatives produced by a single run of the TGDR classifier and thus improve upon parsimony. However, bagging procedure cannot solve this issue completely as shown by the simulation studies [14]. In addition, its computable and theoretical complexity to a clinician unavoidably raised many concerns. In this paper, we proposed to combine a visualization method called Radial Coordinate Visualization (RadViz) [15] with a suitable classifier to discriminate these two NSCLC subtypes, basing on patients' gene expression profiles.

Among many data visualization methods, prevalent means for exposing interesting patterns graphically, Radviz can display data with three or more attributes in a 2-dimensional projection. Radviz has been demonstrated as a complement to computational methods for inference of classification models to search for biologically interesting patterns [16]. Moreover, graphic representation is more easily understandable and implementable for a clinician than statistical methods and/or mathematic equations. However, if using alone to classify new samples, the corresponding posterior probability of the class memberships is absent in a Radviz, thus many performance statistical metrics such as generalized Brier score (GBS) [17] are not computable. Here, we illustrate Radviz can be a useful tool to improve on parsimony without any suffering in accuracy when being combined with a suitable classifier.

## Results

### Synthesized data

In order to evaluate the empirical performance of RadViz in terms of selecting informative features and eliminating irrelevant features, we used the simulations presented in one previous study [14]. However, the assignment of the class membership was accorded to pre-determined logit functions *f*. Specifically, there were 3 classes, 71 samples, and 384 features. The logit functions for class 2 and 3 having class 1 as reference were given by following relationship for three synthesized datasets,

**Simulation 1.**

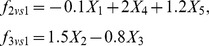



where the logits for class 2 and 3 are two functions with different parameters and the number of relevant features is 5. This is exactly one simulated data used in Tian et al [14].

**Simulation 2.**

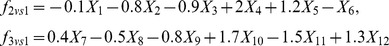



where the logit for class 2 depends only on features X_1_∼ X_6_ while the logit for class 3 depends on features X_7_∼ X_12_. The number of relevant features is 12, several features more than the usually considered number of features in a RadViz projection (usually 3–8 features).

**Simulation 3.**

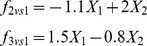



where the number of relevant features is two, less than the minimum number of features in RadViz projections. All parameters used in the simulations were simulated from a uniform distribution on the interval of 0 to 2, i.e., unif (0∼2). By this means, the true relevant features are known and whether RadViz can select them correctly can be investigated.

Upon the simulated data, 50000 RadViz projections were evaluated. Then we locally optimized best projections for 10000 times. The choice of the number of RadViz projections appeared to have little impact on the final results. We obtained very similar results with different numbers, i.e., 10000, 20000, 30000, 40000, 50000, 80000, and 100000. Because the study in [16], which had smaller sample size and more features, used 100000 RadViz projections, we fixed the parameter at 50000 in our study.

This procedure had been run by varying the maximum number of features in a projection from 3 to 8. The obtained best projection for each run was listed in Table 1. Moreover, the features were ranked based on the frequencies they had appeared in all considered RadViz projections. Those most frequently appearing features (All features were output until the last true relevant feature was selected) were also given in Table 1. In summary, RadViz projections can successfully identify the true relevant features with an optimal subset of reasonable small size, even in the case where irrelevant features were highly correlated with relevant ones.

**Table 1:** Results of simulation studies.

Max # of features	Simulation 1 (selected features/VizRank Score)	Simulation 2 (selected features/VizRank Score)	Simulation 3 (selected features/VizRank Score)
3	X4, X18, X3 (89.12%)	X10, X38, X5 (68.67%)	X1, X2, X75 (73.35%)
4	X4, X3, X18, X9 (90.44%)	X5, X10, X3, X38 (75.35%)	X1, X173, X2, X7 (75.78%)
5	X9, X2, X12, X3, X5 (93.06%	X5, X10, X3, X38 (75.35%)	**X170, X1, X7, X2, X173 (78.34%)**
6	X5, X4, X2, X12, X11, X3 (94.22%)	X6, X11, X2, X1, X10, X5 (78.04%)	X1, X28, X3, X32, X2, X83 (78.09%)
7	**X5, X3, X11, X12, X9, X18, X4 (96.08%)**	X5, X4, X11, X38, X21, X3, X10 (79.75%)	X170, X1, X7, X2, X173 (78.34%)
8	X3, X5, X4, X6, X9, X2, X11, X12 (94.15%)	**X7, X4, X11, X2, X3, X12, X10, X6 (80.81%)**	X170, X1, X7, X2, X173 (78.34%)
Frequent ones	X3, X4, X9, X12, X5, X11, X2, X16, X6, X131, X18, X1	X11, X16, X10, X6, X1, X3, X131, X2, X4, X7, X38, X5, X8, X9, X328, X12	X1 X4, X72, X338, X3, X173, X2

### Real data

**Figure 1:**
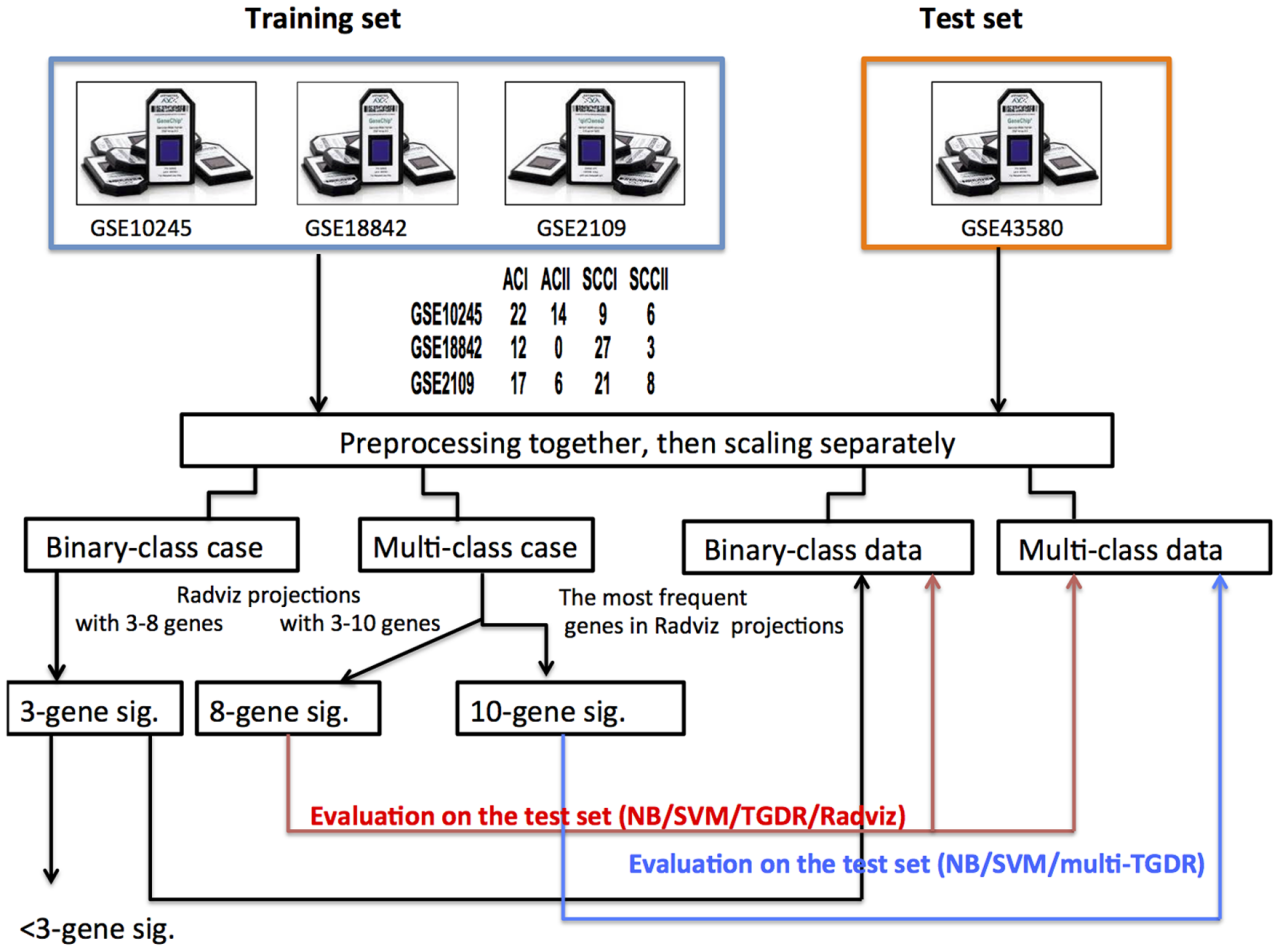
Study flowchart.

The study schema is shown in Figure 1. First, we show the results on AC and SCC subtype-classification. Then we show the results on multi-class classification by considering both subtypes and their respective stages. For two-class Radviz constructions, the numbers of the maximum features in projections varied from 3 to 8, the one obtaining the best VizRank Score [18] (see the method section for the details) was presented. Similarly, for multi-class cases the number of maximum features in projections ranged from 3 to 10, the one with the best VizRank score was tabulated.

### On AC/SCC subtypes (two-class case)

For the winner of the SBV LC subtask [19], referred to as Ben-Hamo's study herein, *keratin 5* (KRT5) is the only gene used to discriminate SCC and AC with an astounding misclassification rate on the test set of 15.3% (23/150). TGDR analysis selected 20 genes as shown in our previous work [8] with a slightly worse prediction error of 16%. Nevertheless, the parsimony of hierarchical-TGDR model obviously lags behind. TGDR is an embedded feature selection algorithm, which means it simultaneously selects the potential informative features with classifier construction. Even combined TGDR with Bagging, our previous study on the simulated data showed that the inferiority of parsimony does not eliminate completely [14]. Alternative methods that can ensemble with TGDR/multi-TGDR to filter out the false positives are in demand. Radviz is one of such methods given its goal is to usually use 3–8 features to show a good separation among classes.

With the aid of Radviz, the best projection obtained from the training set, which ignores the stages, consisted of 3 genes. They are *keratin 5* (KRT5), *RAR-related orphan receptor C* (RORC), and *melanoma antigen family A 4* (MAGEA4). Interesting, both KRT5 and MAGEA4 were selected by the 23-gene signature of Ben-Hamo's study. On these 3 genes, the performance of different classifiers was evaluated and their respective statistics are shown in Table 2A.

**Table 2:** Performance metrics of classifiers on the lung cancer test set (AC and SCC subtype classification).

	The data used (Total # of samples)	N# of Genes	Error (%)	GBS (0)	BCM (1)	AUPR (1)
**Ben-Hamo's study**	GSE10245, GSE18842, GSE31799 (151, 81AC, 70SCC)	1	15.3	NA	NA	NA
**TGDR**	GSE10245, GSE18842, GSE2109, GSE31908 (175, 100AC, 75SCC)	20	16	0.1153	0.8325	0.9416
**A. Radiz on 3-gene signature selected by AC and SCC subtype classification**						
**Radviz alone**	GSE10245, GSE18842, GSE2109 (only stage I &II, 145, 71AC, 74SCC)	3	16.67	–	–	–
**Radviz +TGDR**	GSE10245, GSE18842, GSE2109 (145)	3	14.67	0.2360	0.5144	0.8917
**Radviz+naïve Bayes**	GSE10245, GSE18842, GSE2109 (145)	3	13.33	0.1260	0.8447	0.8908
**Radviz+SVM**	GSE10245, GSE18842, GSE2109 (145)	3	13.33	0.1208	0.6974	0.8978
**B. Radiz on 8-gene signature selected by subtype & stage classification**						
**Radviz alone**	GSE10245, GSE18842, GSE2109 (145)	8	14	–	–	–
**Radviz +TGDR**	GSE10245, GSE18842, GSE2109 (145)	8	13.33	0.1061	0.8271	0.8935
**Radviz+naïve Bayes**	GSE10245, GSE18842, GSE2109 (145)	8	12.67	0.1191	0.8719	0.9067
**Radviz+SVM**	GSE10245, GSE18842, GSE2109 (145)	8	14	0.1029	0.7983	0.9211

NA: not available. –: not computable because no posterior probabilities were provided.

#### ROC curves

**Figure 2:**
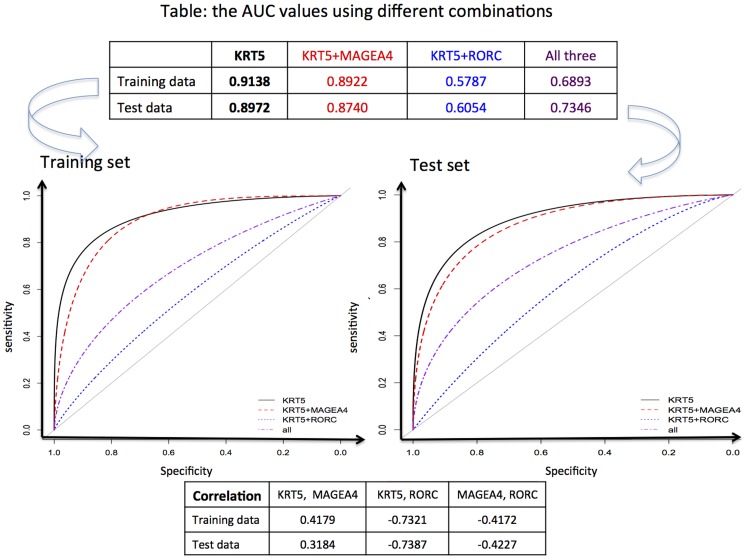
ROC curves for 3-gene signature combinations. The signature of KRT5 alone has the best AUC values on both training and test sets.

**Figure 3:**
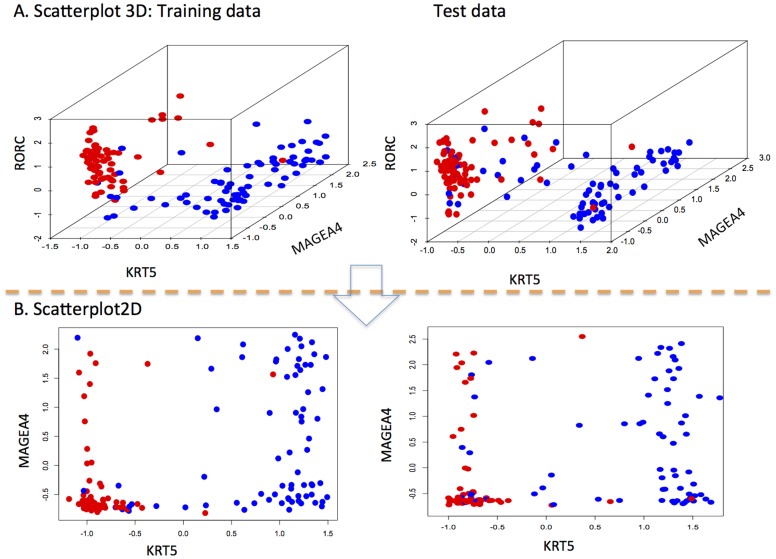
Scatterplots on the training data and test data. A. 3D scatterplots with KRT5 on x-axis, MAGEA4 on y-axis, and RORC on z-axis. B. 2D scatterplots with KRT5 on x-axis, MAGEA4 on y-axis. From these scatterplot, it is obvious that KRT5 alone can discriminate AC and SCC samples apart on both training and test sets.

Considered that the minimum number of features in a Radviz is three, we additionally evaluated on the different combinations of these 3 genes to find the optimal subset. The respective ROC curves were plotted in Figure 2. It provided some justification that KRT5 only can tell AC and SCC apart perfectly as shown in Figure 3. Also, Pearson's correlation coefficients among feature pairs were computed, which indicated that KRT5 and RORC were highly negatively correlated in both training data set and test set. Notably, the AUC value with RORC only was secondary to that of KRT5 only. KRT5 had been consistently identified [8], [16], [19] to explain the difference between SCC and AC, which suggested that it was highly likely to be the true ‘driver’ [20]. Therefore, RORC might be a ‘passenger’ [20] gene, meaning it might be a downstream regulated gene controlled by KRT5 located upstream. Upon a RNA-seq data, Radviz was used to select relevant features. Again, KRT5 was appeared in the final model. The results were presented in File S1. In summary, KRT5 might drive in discrimination of AC and SCC. The analyses conducted on data using both microarray and RNA-seq technologies support this conclusion. It is recommended that a new diagnostic kit on KRT5 be designed to complement the current-used gold standard and to aid the precise and easy diagnosis of these two NSCLC subtypes.

#### The identification of erroneously labelled samples

In the test set, there are six samples that might be wrong-labelled with extremely high misclassification rates in the SBV LC subtask, as shown by the Ben-Hamo's study. Here, we evaluated all considered methods herein and found out almost all of those methods including RadViz + SVM/TGDR/NB or RadViz alone indicated opposite labels to the given ones for these six samples, as shown in Table 3. Indeed, whether these six samples have been mislabelled or not deserves further investigation.

**Table 3:** Might-be wrongly labeled samples identified by Ben-Hamo's study.

ID	Label	Overall SBV misclassification rate	Methods indicating opposite labels
115	AC	84%	All eight methods
19	SCC	86%	All except Radviz alone on 3 gene signature
100	SCC	88%	All except Radviz alone on 3 gene signature
3	SCC	90%	All eight methods
70	SCC	76%	All except Radviz alone on 3 gene signature
9	SCC	88%	All eight methods

### On both subtypes and stages (multi-class case)

**Figure 4:**
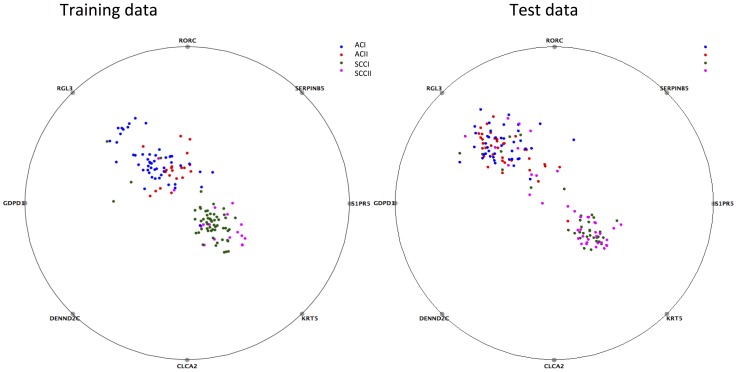
RadViz plots using 8-gene signature on the training data and test data. From these plots, it is observed that AC and SCC samples can be discriminated with a reasonable size of misclassification rate on both training and test sets. However, the discrimination between different stages within each subtype is not achieved on both training and test datasets.

Another Radviz was plotted by considering both subtypes (i.e., AC and SCC) and stages (i.e., stage I and II) strata. The best projection involved 8 genes, including KRT5 and RORC also selected by the two-class Radviz best projection. With these 8 genes, the performances of different classifiers were evaluated. The results were shown in Table 4B, from which we observed that there was no obvious winner. Multi-TGDR had the best *Generalized Brier Score* (GBS) and SVM had the best *Area Under the Precision-Recall Curve* (AUPR) while naïve Bayes outperformed in terms of *Belief Confusion Metric* (BCM). Moreover, the performance of these 8 genes on SCC and AC classification was evaluated and the results were shown in Table 2B. Using 8 genes on the histology subtype classification, the respective statistics showed slightly superiority. It is observed that AC and SCC samples can be discriminated with a reasonable size of misclassification rate on both training and test sets. However, no perfect discrimination between stages I and II within each subtype has been achieved on both training and test datasets, as shown in Figure 4.

**Table 4:** Performance metrics of classifiers on the lung cancer test set (subtype and stage classification).

	N# of Genes	Error (%)	GBS (0)	BCM (1)	AUPR (1)
**A. On classifiers**					
Ben-Hamo's study	23	49.3	NA	0.48	0.46
Hierarchical-TGDR	66	53.3	0.3736	0.4401	0.4709
Pairwise Coupling	158	54	0.3794	0.4371	0.4010
Multi-TGDR local	83	54	0.3579	0.4210	0.4681
Multi-TGDR global	60	54	0.3524	0.4164	0.4685
**B. Radviz on 8-gene signature selected by subtype and stage classification**					
Radviz + multi-TGDR	8	54.7	0.3423	0.4137	0.4557
Radviz+ naïve Bayes	8	54.7	0.4104	0.4437	0.4494
Radviz+SVM	8	54	0.3654	0.4137	0.4562
**C. Radviz on the most frequently selected features**					
Radviz + multi-TGDR	10	53.3	0.3215	0.4269	0.4710
Radviz+ naïve Bayes	10	55.3	0.4256	0.4503	0.4573
Radviz+ SVM	10	54.7	0.3516	0.3612	0.4815

NA: not available.

An alternative way of utilizing Radviz seamlessly is to consider the most frequent selected genes by all projections and then to find the potential informative features. In this method, the features were ranked based on the frequencies they had appeared in all RadViz projections. Those most frequently appearing features were considered. Among them, we selected a subset comprised of 7 over-expressed genes in SCC and 3 over-expressed genes in AC. Using these 10 genes, the performances of different classifiers were re-evaluated and the respective statistics were listed in Table 4C. This time multi-TGDR obtained a performance comparable to that of hierarchical-TGDR. Interestingly, there was a big overlap between 10 genes and those selected by hierarchical-TGDR and other methods, including KRT5. Most of them were biologically and clinically meaningful. For example, *desmocollin 3* (DSC3) and *desmoglein 3* (DSG3) had been recently justified as valuable in classification of NSCLC subtypes [21] while *Chloride channel accessory 2* (CLCA2) had been recognized as a tumorigenesis gene.

## Discussion

Radviz makes two implicitly false assumptions by evenly placing features around the circle [22], which are 1) these features are uncorrelated, and 2) these features are of equal importance. Being used alone as a classification method, Radviz does not show any superiority over other methods with respect to predictive accuracy let alone many other statistics are not computable there. However, as a complement to other classifiers, it may serve to optimize the model parsimony. Upon the selected features, the ensembles of Radviz and NB/SVM/TGDR have comparable predictive performance to other methods such as hierarchical-TGDR.

From our analysis, it is found that TGDR/multi-TGDR methods might be in favor of AUPR and GBS but less preferable to BCM. Notably, TGDR and multi-TGDR frameworks ignore the co-expression features among genes. Thus those genes that belong to the same/similar pathways or networks tend to end up the final model. To tackle with this limitation, Ma and Huang [23] proposed a new algorithm called clustering-TGDR. However, extensions of clustering-TGDR to multi-class cases have not been addressed, which is one of our future works. When combing with Radviz, TGDR/multi-TGDR methods do not show much superiority over other classifiers.

Finally, the poor performance of multi-class classifiers may be due to the lack of molecular signature difference in different stages of NSCLC. If this is true, it means that most of the selected features are purely random noises. The fact that no team in the SBV LC subtask can separate the respective stages within each subtype shed some evidence on this conjecture. Nevertheless, our observation is that the discrimination of ACI and ACII seems to be easier compared to that of SCCI and SCCII. In **File S1**, those classifiers were applied to another independent test set. The good separation between AC and SCC samples and the poor performance of multi-class classifiers were consistently observed, which provides some justification on the validity and robustness of our study here.

Our proposal of using Radviz to do feature selection, especially using the most frequently selected features by thousands of Radviz best projects, is a unique feature. Other visualization methods such as a heat-map cannot cope with feature selection, thus Radviz has its advantageous merit. Additionally, even though visualization ensembles were originally proposed upon microarray experiments, its broad applications to other “omics” data are out of question as shown by its applications to a RNA-seq data and to a metabolomics data in **File S1**.

On the complicated LC classification task, the ensemble of Radviz and a classifier can obtain perfect model parsimony with reasonable predictive performance. With this proposal the segmentation of early stages in two LC major subtypes is still unachievable, however, we believe more novel algorithms or novel ensembles of existing methods will be developed by quantitative researchers in the near future. Then using these benchmark data sets, the comprehensive understanding of the underlying mechanism associated with NSCLC subtypes and respective stages becomes highly possible.

## Materials and Methods

### Microarray data

The lung cancer microarray experiments under consideration included all chips in the Gene Expression Omnibus' (GEO) repository series GSE10245, GSE18842, GSE2109, GSE31908, and GSE43580 (test set) all of which were hybridized on Affymetrix HGU133 Plus 2.0 chips.

### Pre-processing procedures

The raw Affymetrix data (CEL files) of all lung cancer data sets were downloaded from the GEO repository, and expression values were obtained using the fRMA algorithm [24]. Then the training data was normalized to the target distribution of the testing data set. To address the batch effects from different experiments, the COMBAT algorithm (http://www.bu.edu/jlab/wp-assets/ComBat/Abstract.html) was used to adjust for the combined expression values for these studies because a comprehensive evaluation on several commonly-used batch-effect adjustment methods showed that COMBAT outperformed others overall [25].

Moderated t-tests (limma package)were conducted to identify differentially expressed genes (DEGs) with cutoffs for False Discovery Rate (FDR) and fold change as 0.05 and 2, respectively. When there were multiple probe sets representing the same gene, the one with the largest fold change was chosen. Finally, expression values were further centralized and normalized to have a mean of 0 and a variance of 1 for both training data and test data, respectively. The resulting normalized expression values for 676 unique genes were fed into the downstream classification analysis.

#### Radviz & VizRank

In a Radviz, the features such as genes are presented as anchor points spaced around the perimeter of a circle while samples are as points inside the circle. The position of one sample is determined by a metaphor from physics, saying each point is held in place with springs that are attached at the other end to the feature anchors. The stiffness of each spring is proportional to the value of the corresponding feature and the point ends up at the position where the spring forces are in equilibrium. Therefore, subjects that are close to a set of feature anchors have high values on these features than on the others.

In order to obtain a clear and good separation among different classes using just several features, Radviz needs to search over a myriad of possible combinations. Therefore the search is tedious. To automatically solve this problem, an approach called VizRank had been proposed by [18], which scores the visualization projects according to the degree of class separation and investigates over possible projection candidates to find those with the highest scores. Briefly, VizRank implements a heuristic search, which saves on the computing time. The features are ranked using signal-to-noise ratio and a subset of the features is randomly chosen favoring features with higher ranks. By doing this, genes with more information about the given classification problem are more likely to be selected in a Radviz projection. Upon a selected gene subset, VizRank then evaluates exhaustively all possible Radviz projections defined by different permutations of feature anchors on a unit circle.

#### The classifiers

Here, we used Radviz to do the redundant feature elimination, and used an extra classifier to classify samples and to compute posterior probabilities. Those classifiers were described briefly as follows.

### TGDR and Multi-TGDR

As mentioned in the Introduction section, the TGDR framework was presented and described by Ma and Huang [10] in details. For the detailed descriptions on multi-TGDR frameworks and how the tuning parameters k and τ regularize the sparseness of the final models, our previous work [12], [14] is referred. Two things worthy to be mentioned about multi-TGDR frameworks are 1) when multi-TGDR frameworks are simply used as classifiers, i.e., they only serve to estimate the beta parameters of a pre-determined feature set without implementing the feature selection, multi-TGDR global and multi-TGDR local correspond to the same thing since the tuning parameter τ is set as zero. 2) When the number of the classes is two, both multi-TGDR frameworks collapse back into TGDR.

### Naïve Bayes

A naïve Bayes (NB) classifier is a simple probabilistic classifier. Based upon Bayes' theorem, it makes an independence assumption, which assumes that features are independent giventhe class. The performance of NB on data sets with redundant features can be improved by removing such features usually with a forward search strategy being used with NB since it can immediately detect dependencies as many redundant features being added.

### Support Vector Machine (SVM)

Simply put, SVM [26] used a kernel function to implicitly map data to ahigh dimensional space. Then, it constructed the maximum margin hyperplane by solving an optimization problem on the training data. SVMs have been shown to work well for high dimensional microarray data sets, especially on two-class classifications [27].

#### Statistical Metrics

According to [5], considering a single metric as only standard on evaluation of an algorithm tends to produce bias, and an algorithm may be erroneously claimed as superiority if a metric favouring it were chosen. Thus we use four metrics i.e., *Belief Confusion Metric* (BCM), *Area Under the Precision-Recall Curve* (AUPR), *Generalized Brier Score* (GBS), and predictive error rate on the test set, to evaluate the performance of the combinations between Radviz and different classifiers more precisely and rigorously.

GBS is described in details in our previous work [8], [12]. In principle, the closer it is to zero the better a model is. BCM and AUPR are among three metrics used by SBV challenge. Why those metrics were chosen in SBV challenge is explained in [7]. The definition and interpretation of BCM and AUPR are available in the SBV homepage (http://www.sbvimprover.com/sites/default/files/scoring_metrics.pdf).

#### Statistical language and packages

The statistical analysis was carried out in the R language version 3.0 (www.r-project.org) and packages such as limma were from the Bioconductor project (www.bioconductor.org). The analysis using visualization methods including RadViz and VizRank was conducted in the Orange software, version 2.7 (www.orange.biolab.si).

## Supporting Information

File S1 **Supplementary Materials.**
(DOCX)
