# Supplementary material for: Visualization-Aided Classification Ensembles Discriminate Lung Adenocarcinoma and Squamous Cell Carcinoma Samples Using Their Gene Expression Profiles
Source: PLoS One. 2014 Oct 15;9(10):e110052. doi: 10.1371/journal.pone.0110052 (PMC4198193; doi:10.1371/journal.pone.0110052)
Supplement: File S1 — Supplementary Materials. (DOCX) [file pone.0110052.s001.docx]

**Supplementary materials:**

**Validation on another test set**

To test that the stability and robustness of the proposed visualization classification ensembles on feature selection and parameter estimation, another independent microarray data (Accession No. GSE50081 in GEO) was used as a test set for more validation. The arrays were hybridized on Affymetrix HGU133 Plus 2.0 chips and there were 127 AC and 42 SCC samples, respectively.

The raw Affymetrix data (CEL files) were downloaded from GEO repository and expression values were obtained using the *GCRMA* [1] algorithm. Data normalization across samples was carried out using quantile normalization and the resulting expression values were log_2_ transformed. Then extra scaling process was applied to standardize the means of those expression values as zeros and variances as ones.

As shown in Supp. Table 1 and Supp. Table 2, even though this test set was preprocessed and normalized completely separately from the training set and the first test set, both 3-gene signature and 8-gene signature for the binary classification can discriminate AC and SCC samples apart with decent accuracy. Meanwhile, the near random guess predictive performance of all multi-class classifications using either 8-gene signature or 10-gene signature is also observed. Another consistent observation is that no superiority of one particular classifier can be established over all performance statistics.

**Applications to other “omics” data**

Although in the article the proposed visualization ensembles were applied to a microarray data, it can be applied to other “omics” data with no need of any nontrivial modification. Here, we use one metabolomics data and one RNA-seq data to demonstrate such applications are out of question.

***On metabolomics data***

Over the last decade, metabolomics has evolved into a mainstream enterprise utilized by many laboratories globally. Given metabolomics data analysis is less standardized compared to other “omics” data analysis due to its complexity, many of the existing feature selection algorithms have not been explored and implemented in such data analyses and only a few algorithms have been proposed to specifically analyze such data [2]. Here, the proposed classification ensembles were applied to a metabolomics data, which was analyzed by us previously [3] using multi-TGDR algorithm. The study included 30 patients with cirrhotic liver disease, 70 patient with hepatocellular carcinoma (HCC), and 31 healthy volunteers. There were 384 metabolites under consideration.

For the detailed descriptions of the experimental data and the preprocessing steps on handling the raw data, Tian et al [3] is referred. One limitation of this analysis is that no independent test sets are available, however, it has no effect on our goal of demonstrating the broad application range of the proposed visualization ensembles. The results were presented in Supp. Table 3. Among the 8 selected metabolites by Radviz, there were 5 of them selected by multi-TGDR algorithms as well. As shown by the simulation analyses [3], the negative positive rates of multi-TGDR algorithms are high even after the specific adjustment being made by bagging algorithm [4]. Based on this, it is recommended that special attention on these 5 metabolites be given for their potential role as relevant markers. A comprehensive investigation on this topic is beyond the scope of this article.

***On RNA-seq data***

More relevantly, we applied the ensembles to a RNA-seq data set with 489 AC and 488 SCC samples downloaded from The Cancer Genome Atlas (<https://tcga-data.nci.nih.gov/tcga/>). The primary objective here is to explore the comparability of different omics technologies on the identical classification task. Counts-per-million (CPM) values were calculated and log_2_ transformed by the voom function [Add a reference: Law, C.W., Chen, Y., Shi, W., and Smyth, G.K. (2014). Voom: precision weights unlock linear model analysis tools for RNA-seq read counts. Genome Biology 15, R29.] in the limma package [6]. Then the whole data set was randomly divided into two equal-sized subsets, one serving as the training set and the other as the test set. On the training set, the voom function in the limma package was used to identify differentially expressed genes (DEGs). There were 502 up-regulated and 457 down-regulated genes, respectively. Those DEGs were fed into Radviz, which selected ALPK3, MACC1 and KRT5 as relevant genes.

The results were presented in Supp. Table 4. The performance statistics were better than those calculated on the microarray test sets, this may be due to the adequately large sample size, more precise measurement of gene expression by RNA-seq, and the inclusion of advanced cancer cases. In summary, KRT5 may serve as a diagnostic marker to discriminate two major subtypes of NSCLC apart. The analyses conducted on data from both microarray and RNA-seq technologies support to this conclusion.

**Reference:**

1. Wu Z, Irizarry RA, Gentleman R, Martinez-Murillo F, Spencer F (2004) A Model-Based Background Adjustment for Oligonucleotide Expression Arrays. J Am Stat Assoc 99: 909–917.

2. Noble WS, MacCoss MJ (2012) Computational and Statistical Analysis of Protein Mass Spectrometry Data. PLoS Comput Biol 8: e1002296.

3. Tian S, Chang HH, Wang C, Jiang J, Wang X, et al. (2014) Multi-TGDR, a multi-class regularization method, identifies the metabolic profiles of hepatocellular carcinoma and cirrhosis infected with hepatitis B or hepatitis C virus. BMC Bioinformatics 15: 97.

4. Breiman L (1996) Bagging predictors. Mach Learn 24: 123–140.

5. Robinson MD, McCarthy DJ, Smyth GK (2010) edgeR: a Bioconductor package for differential expression analysis of digital gene expression data. Bioinformatics 26: 139–140. doi:10.1093/bioinformatics/btp616.

6. Smyth GK (2005) limma: Linear Models for Microarray Data. Bioinformatics and Computational Biology Solutions Using R and Bioconductor. pp. 397–420. doi:10.1007/0-387-29362-0_23.

**Supp. Table 1. Performance metrics of two-class classifiers on GSE50081**

|  | **N# of Genes** | **Error** (%) | **GBS** (0) | **BCM** (1) | **AUPR** (1) |
| --- | --- | --- | --- | --- | --- |
| **A. Radiz on 3-gene signature selected by AC and SCC subtype classification** | | | | | |
| **Alone** | 3 | 21.30 | -- | -- | -- |
| **TGDR** |  | 14.79 | 13.70 | 75.18 | 83.17 |
| **Naïve Bayes** |  | 15.98 | **13.64** | **84.63** | **85.08** |
| **SVM** |  | **13.61** | 13.01 | 68.39 | 83.37 |
| **B. Radiz on 8-gene signature selected by subtype & stage classification** | | | | | |
| **Alone** | 8 | 21.89 | -- | -- | -- |
| **TGDR** |  | **8.88** | **10.03** | 81.83 | 84.94 |
| **Naïve Bayes** |  | 15.98 | 15.31 | **84.49** | **87.94** |
| **SVM** |  | 13.61 | 13.55 | 65.97 | 81.00 |

--: Not computable because no posterior probabilities were provided.

**Supp. Table 2. Performance metrics of multi-class classifiers on GSE50081 (subtype and stage classification)**

|  | **N# of Genes** | **Error** (%) | **GBS** (0) | **BCM** (1) | **AUPR** (1) |
| --- | --- | --- | --- | --- | --- |
| **A. Radviz on 8-gene signature selected by subtype and stage classification** | | | | | |
| Radviz + multi-TGDR | 8 | 42.60 | 0.3047 | 0.3990 | 0.3957 |
| Radviz+ naïve Bayes | 8 | 44.97 | 0.3248 | **0.4482** | **0.4664** |
| Radviz+SVM | 8 | **39.64** | **0.2935** | 0.3884 | 0.4560 |
| **B. Radviz on the most frequently selected features** | | | | | |
| Radviz + multi-TGDR | 10 | 37.87 | **0.2852** | 0.4153 | **0.4315** |
| Radviz+ naïve Bayes | 10 | 49.11 | 0.3715 | **0.4296** | 0.4086 |
| Radviz+ SVM | 10 | **37.28** | 0.2907 | 0.3645 | 0.4194 |

**Supp. Table 3. Performance metrics on the metabolomics data**

|  | **Error (%)** | **GBS (0)** | **BCM (1)** | **AUPR (1)** |
| --- | --- | --- | --- | --- |
| Radviz alone | 3.82 | -- | -- | -- |
| Radviz + multi-TGDR | 2.29 | 2.03e-02 | 93.43 | 99.11 |
| Radviz+ naïve Bayes | 8.40 | 7.15e-02 | 93.22 | 98.12 |
| Radviz+ SVM | **0** | **2.37e-03** | **95.61** | **99.46** |

--: Not computable because no posterior probabilities were provided.

**Supp. Table 4. Performance metrics on the NSCLC RNA-seq test data**

|  | **Error (%)** | **GBS (0)** | **BCM (1)** | **AUPR (1)** |
| --- | --- | --- | --- | --- |
| Radviz alone | 5.74 | -- | -- | -- |
| Radviz +TGDR | 5.74 | 4.82e-02 | 88.88 | 97.25 |
| Radviz+ naïve Bayes | **5.12** | **4.44 e-02** | **94.49** | 96.73 |
| Radviz+ SVM | 5.74 | 7.12e-02 | 76.56 | **97.57** |

--: Not computable because no posterior probabilities were provided.
